# Ecological Correlates of Ecological Specialization of Avian Communities in University Campuses of China

**DOI:** 10.3390/biology14050570

**Published:** 2025-05-19

**Authors:** Ling-Ying Shuai, Di Meng, Wan-Lan Ma, Jing-Wen Bai, Yue Luo, Yu-Xin Luo, Zhu-Cheng Gao, Hao Zhu, Zhu-Qin Long

**Affiliations:** 1 College of Life Sciences, Huaibei Normal University, Huaibei 235000, China; mengdi204@163.com (D.M.); mawanlan622@163.com (W.-L.M.); baijingwenlip@163.com (J.-W.B.); lyly20041018@163.com (Y.L.); l3260315812@163.com (Y.-X.L.); 15556559901@163.com (H.Z.); 18056942235@163.com (Z.-Q.L.); 2 College of Life Science and Technology, Harbin Normal University, Harbin 150025, China; 13644578677@163.com

**Keywords:** bird, campus, diet, ecological specialization, foraging stratum, urban green space

## Abstract

With the rapid process of urbanization at a global scale, university campuses have been viewed as important urban green spaces for biodiversity conservation. However, little is known about the role of university campuses in protecting ecological specialists, the species usually vulnerable to anthropogenic disturbance. We assessed the associations between several ecological variates and ecological specialization of bird communities across 198 Chinese university campuses. A total of 398 bird species were recorded, including 109 diet specialist species and 104 foraging stratum specialist species. We found that the elevation of campuses was positively related to diet specialist species richness, and the campus area was positively related to foraging stratum specialist species richness. NDVI was positively associated with the community-wide foraging stratum specialization index, but negatively associated with the community-wide diet specialization index. Our results suggest that campuses with larger areas or located at high elevations play an important role in maintaining ecological specialization of bird communities.

## Simple Summary

Ecological specialists are species with a narrow niche and are usually vulnerable to disturbance and environmental changes. As a special type of urban green space, university campuses often act as biodiversity hotspots in cities, providing essential refuges for many species. It is thus important to understand whether university campuses would also help to maintain ecological specialists. Based on a citizen science dataset, we explored the spatial distribution and ecological drivers of ecological specialization of bird communities across 188 university campuses of China. Among the 398 species recorded in these campuses, 109 and 104 species were categorized as diet and foraging stratum specialist species. The results of modeling highlighted the importance of campus area and elevation in maintaining ecological specialization in birds. However, the effect of net primary production on ecological specialization is relatively complex, with a positive effect on the community-wide foraging stratum specialization index, and a negative effect on the community-wide diet specialization index. As the first continent-wide investigation on the role of university campuses in protecting ecological specialization, our study should provide insights into urban planning and wildlife conservation.

## 1. Introduction

Species with a narrow niche or specialized for a highly limited range of resources (i.e., ecological specialists) are often considered vulnerable to anthropogenic disturbance and environmental changes [1]. Unlike ecological generalists, specialists cannot effectively use alternative resources when their preferred habitats or food resources become scarce [2]. As a result, specialists may lack necessary resilience to cope with rapid environmental changes and are therefore easier to go extinct [3]. The level of specialization is often negatively related to geographic range size [4,5] and often acts as an important predictor of extinction risk [6,7,8]. Moreover, fossil records also suggest that species with a higher level of dietary specialization generally possess shorter species durations [9]. However, ecological specialization has only received limited attention in conservation practice, and has rarely been considered in development of conservation plans [10,11,12].

As a fundamental type of global change, urbanization poses threats to biodiversity and ecosystem productivity through many processes, such as habitat loss, ambient noise, light pollution, and the heat island effect [13,14,15,16]. Compared to generalists, specialists are usually less adapted to city environments, making urbanization especially dangerous for ecological specialists [17,18,19,20]. Nevertheless, even within cities, some urban green spaces (e.g., parks, university campuses, and private gardens) can provide effective refuges for many species and therefore partly alleviate the negative effects of urbanization on biodiversity [21]. Among these, university campuses deserve special attention, as they are usually large in area, widely distributed, and often possess considerably high biodiversity [21]. Moreover, people in campuses (mostly students and teachers, among which there are many naturalists and conservationists) are often well educated and more willing to participate in field surveys, nature conservation, or other environment-friendly practices [21]. As a result, university campuses may create a great opportunity for monitoring and conservation of urban biodiversity [21,22]. However, it remains poorly known whether these places also help to maintain ecological specialists, the species considered most severely threatened by urbanization.

As a country with extremely high levels of biodiversity [17], China has experienced rapid urbanization in recent decades [18]. This process has not only caused considerable threats to many species, but also brought about significant changes in biodiversity pattern [19,20]. Meanwhile, China possesses a large number of university campuses (including colleges), covering a considerable proportion of urban area [21]. Compared to other urban areas, these campuses often provide better habitats for wildlife and possess higher biodiversity. For example, 29% (393 species) of the overall Chinese bird species can be found in a total of 38 Chinese universities [21]. Moreover, university campuses are home for many naturalists, researchers, and conservationists, making it convenient to conduct comprehensive investigations on biodiversity in campuses. Therefore, university campuses in China provide an ideal study system to assess the role of urban green spaces in promoting biodiversity conservation.

In this study, we aimed to explore the distribution of ecological specialization of bird communities in Chinese university campuses, as well as its underlying ecological drivers. Two types of specialization were studied, namely dietary specialization and foraging stratum specialization. For each type of specialization, we focused on two indicators, i.e., richness of avian specialist species and community-wide specialization index (hereafter CSI) within each university campuses. Specifically, we had several predictions to test: (1) richness of specialist species should increase with increasing campus area, due to the generally positive species–area relationship [22] and the fact that specialist species are a subset of overall species within a community [10]; (2) richness of specialist species and CSI should decrease with increasing human population density, because specialists are usually considered more vulnerable to anthropogenic disturbance; (3) campuses with higher primary productivity (denoted by normalized differential vegetation index in this study, hereafter NDVI) tend to possess more specialist species, because these places are likely to possess more abundant and more diverse resources, enabling more specialist species to survive; and (4) campuses located at higher elevation tend to possess more specialist species, since high-elevation regions are generally less populous, so that specialist species would be less exposed to anthropogenic disturbance. Our study should provide insights for a deeper understanding of the value of urban green spaces in wildlife and biodiversity conservation.

## 2. Materials and Methods

### 2.1. Data on Bird Communities

The data on avian communities used in this study were collected from the dataset of a recently published research [23]. The original dataset was based on an extensive literature search in September 2022, using 64 relevant papers found in several major scholar databases (ISI Web of Science, Google Scholar, and the China National Knowledge Infrastructure) and 1280 reports collected from the China Bird Recording Center. The original dataset includes both birds recorded in 287 university campuses from 75 cities and those recorded in the nearby natural reserves, resulting in a total of 745 avian species recorded in 75 cities. For this research, we excluded data from natural reserves, as well as data from campuses which were seemingly inadequately sampled (with a checklist including less than 10 bird species per campus) or whose exact locations could not be identified. We also excluded those bird species not reliably recorded in the campuses from the original dataset, with 398 avian species (recorded in 188 university campuses located in 65 cities) remaining in the final check list (Table S1).

### 2.2. Assessment of Ecological Specialization

We assessed the distribution of two types of ecological specialization, namely dietary and foraging stratum specialization (Table S2). Information on diet and foraging stratum for each species was obtained from the EltonTraits 1.0 database [24]. In this database, diet composition was described using percentages of 10 major types of food, including invertebrates, vertebrates (endotherm), vertebrates (ectotherm), vertebrates (fish), vertebrates (unknown), scavenger, frugivore, nectarivore, granivore, and folivore. The percentages were determined using a semi-quantitative approach, with scores calculated based on the word order in original sentences in literature that describe the diet [24]. Similarly, foraging stratum composition was expressed with percentages of 7 major stratum, namely foraging below the water surfaces, foraging on or just below water surfaces, foraging on ground, foraging in the understory, foraging in mid to high levels in trees or high bushes, foraging in or just above the tree canopy, and foraging well above trees or any structures [24].

Based on diet and foraging stratum composition, we further assessed the degree of diet and foraging stratum specialization for each species. Following the approaches used by Morelli et al. [25], we adopted the Gini index of inequality to reflect the degree of specialization using the following formula:

$$G=\frac{\sum_{i=1}^{n}\sum_{j=1}^{n}\left[x_{i}-x_{j}\right]}{2n^{2}x^{\prime }}$$

where ‘*x*’ is an observed value (in percentage), ‘*x*′’ is the mean value of the percentages, and ‘*n*’ is the number of values observed.

To assess the overall degree of ecological specialization within a given community, we adopted two indicators for each type of specialization: richness of specialist species and community-wide specialization index (CSI). Following the criterion used by Morelli et al. [10], we defined a species to be dietary or foraging stratum specialist if its relevant Gini index equals to 1, which means that species only used one type of food or only foraged in a single stratum. For each campus, richness of specialist species was therefore denoted by the number of specialist species, and CSI was calculated as the sum of the Gini index divided by the number of all the species detected in that campus.

### 2.3. Environmental Factors

To explore the underlying mechanisms driving the spatial pattern of avian specialization across Chinese university campuses, we considered the following environmental factors: campus area, elevation, NDVI, and human population density (Table S3). We obtained a recent vectograph of China generated on OpenStreetMap (downloaded from Geofabrik with the URL of https://download.geofabrik.de/asia/china (accessed on 26 September 2024) and searched for the polygon representing each campus by its name. We then used ArcGIS 10.2.2 to calculate the area of each campus. Information on elevation, NDVI, and human population density for each campus was collected using ArcGIS 10.2.2 from the relevant grid datasets (data for 2020, with a spatial resolution of 500 m) provided by the Data Center for Resources and Environmental Sciences, Chinese Academy of Sciences (RESDC, https://www.resdc.cn (accessed on 10 July 2024). For each of the grid datasets, if a campus covered several pixels, an average value of these pixels was calculated to represent the relevant environmental value for that campus.

### 2.4. Statistical Analysis

For each of the four response variables related to specialization (diet specialist richness, foraging stratum specialist richness, diet CSI, and foraging stratum CSI), we built a generalized least square linear model (GLS model) with the following explanatory variables: species richness, area, elevation, NDVI, and human population density. Species richness was included because specialist species were subsets of local assemblages and specialist richness was often highly associated with the overall species richness [11]. We also built a GLS model on species richness, using area, elevation, NDVI, and human population density as explanatory variables. For all these GLS models, a Gaussian correlation structure including the latitude and longitude of the centroid of each campus was used to take spatial autocorrelation into account [11,26]. To assess the level of multicollinearity, we calculated variance inflation factors (VIFs) using the ‘vif’ function provided by the R package ‘car’ [27]. Since the level of multicollinearity was generally low (all VIFs were lower than 5), all the explanatory variables were retained in the final model sets.

## 3. Results

**Figure 1:**
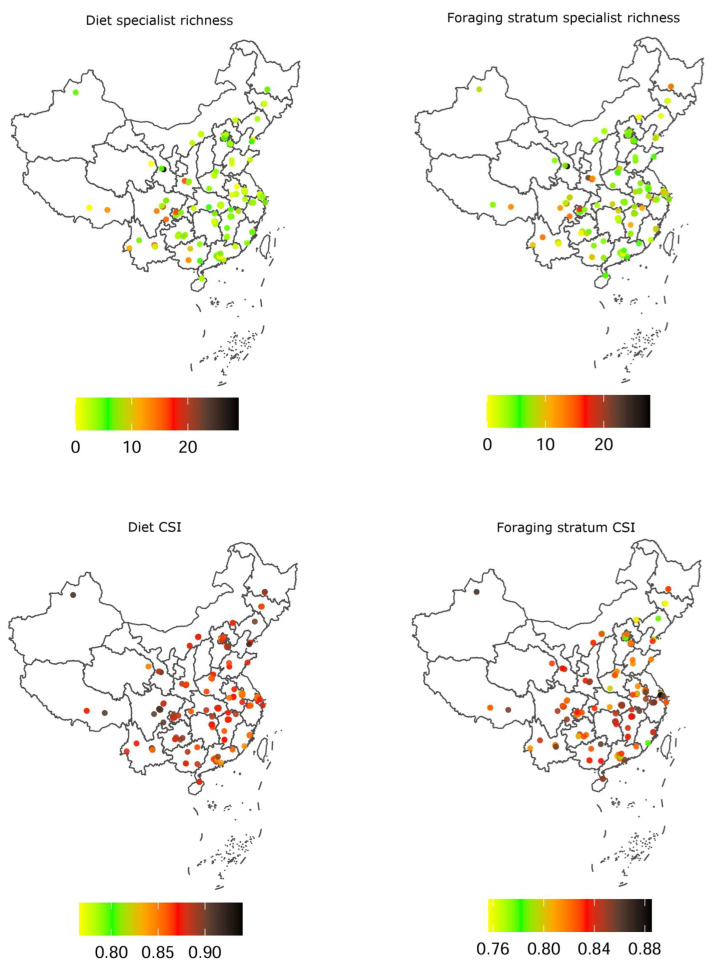
Distribution of ecological specialization among the 188 university campuses included in this study. Dots represent locations of campuses and colors represent values of the indicators of ecological specialization. CSI: community-wide specialization index.

Among the 398 avian species recorded in the 188 university campuses, 109 (27.39%) and 104 species (26.13%) were categorized as diet specialists and foraging stratum specialists, respectively. Among these campuses, species richness ranged from 10 to 96 species (29.18 ± 16.63 species, mean ± SD, and hereafter). Diet specialist richness and foraging stratum specialist richness ranged from 0 to 29 species (4.80 ± 4.26 species) and from 0 to 28 species (6.45 ± 4.44 species), respectively (Figure 1). Diet CSI and foraging stratum CSI ranged from 0.77 to 0.94 (0.87 ± 0.02) and from 0.76 to 0.89 (0.83 ± 0.03), respectively (Figure 1).

**Figure 2:**
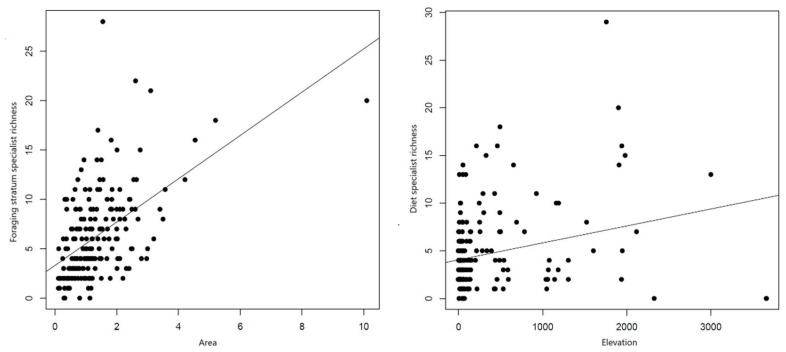
Association between ecological variables and specialist richness. Left: campus area (in km^2^) versus foraging stratum specialist richness. Right: elevation (in m above sea level) versus diet specialist richness.

**Figure 3:**
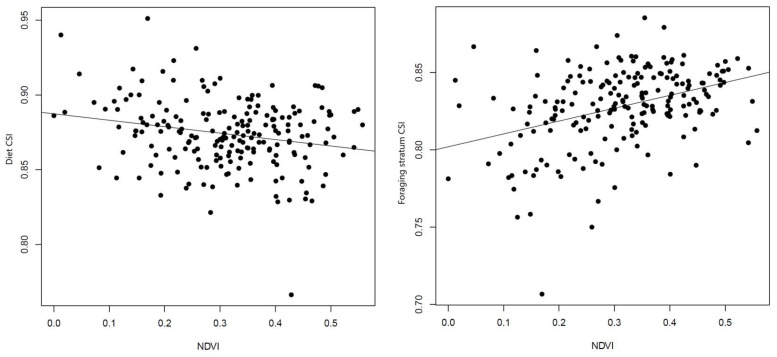
Association between normalized difference vegetation index (NDVI) and community-wide specialization index (CSI). Left: NDVI versus diet CSI. Right: NDVI vs. foraging stratum CSI.

According to the GLS models, species richness was positively related to campus area, NDVI, and elevation (Table 1). As an explanatory variable, species richness was also positively associated with diet specialist richness, foraging stratum specialist richness, and diet CSI (Table 1). We detected a positive relationship between elevation and diet specialist richness, and a positive association between campus area and foraging stratum specialist richness (Table 1; Figure 2). NDVI was positively related to foraging stratum CSI, but negatively related to diet CSI (Table 1; Figure 3). No significant association was detected between human population density and any measurement of ecological specialization (Table 1).

## 4. Discussion

For the first time, we conducted an extensive study on the ecological specialization of bird communities in Chinese university campuses. More specifically, we assessed the associations between four measures of specialization and environmental variables. Generally, campuses located in higher areas tended to possess more diet specialist species and larger campuses tended to possess more foraging stratum specialist species. We also detected significant relationships between NDVI and two community-wide specialization indices, but with different directions between the two types of CSI. Contrary to our prediction, human population density within campuses was positively related to diet specialist richness. Our results highlighted the role of large or high-elevation campuses in protecting avian specialization. Our study should provide valuable information for urban planning and prioritizing urban green spaces for conservation.

Elevation has long been documented as a fundamental driver of changes in various communities [28,29,30,31]. Consistent with a previous study on avian specialization [11], we found that higher elevations were generally related to more diet specialist species. In this study, the 198 university campuses showed a dramatic variation in elevation, ranging from −6 m a.s.l. (Guangzhou University) to 3662 m a.s.l. (Tibet University). This in turn brings about a significant variation in climate and the availability of food resources among campuses. Organisms living at high altitudes usually suffer more severe climates, which may act as an environmental filter so that only those species highly adapted to such climates can be sustained [12]. These species are often specialists. Food resources should also be less diverse in high-elevation areas, so that species specialized for certain food types (i.e., diet specialists) may outcompete generalists (‘jack of all trades and master of none’). Moreover, high-elevation cities are often less populous [32] and less accessible, and species living there may face less severe anthropogenic disturbance. This should in turn allow more specialists to survive in these cities, since specialists are often more vulnerable to anthropogenic disturbance [3,33]. In summary, our results emphasized the role of elevation in driving the ecological specialization of urban birds [11,34].

We found that campus area was positively related to foraging stratum specialist richness. Usually, sites with larger areas tend to possess more types of habitat [35,36], which can provide more foraging niches to accommodate more types of foraging stratum specialists. For example, large campuses (such as Peking University, Wuhan University of Science and Technology, and Guangxi University) often possess one or more lakes with considerable size [37], providing two important foraging stratums (foraging below the water surfaces, and foraging on or just below water surfaces) for many foraging stratum specialist species, such as Common Kingfisher (*Alcedo atthis*), Mandarin Duck (*Aix galericulata*), Ruddy Shelduck (*Tadorna ferruginea*), Cotton Pygmy Goose (*Nettapus coromandelianus*), and Garganey (*Anas querquedula*). By contrast, small campuses often lack such lakes. Moreover, large campuses may also possess more places that are silent and less populous (such as Minghe Park and Jingchun Park in Peking University) [37], providing important refuges for specialists to stay away (at least temporarily) from anthropogenic disturbance.

Although anthropogenic disturbance is a fundamental factor that puts species at risk (and especially ecological specialists), our results suggest that human population density in campus was not significantly associated with most measures of ecological specialization of avian communities. Moreover, we even detected a positive relationship between human population density and diet specialist species richness. The uneven distribution of human population within universities might have contributed to this counterintuitive pattern. In university campuses, the majority of the human population is usually distributed in teaching buildings and dormitories, leaving some regions rarely occupied by people, which is more common in larger campuses as documented above. Moreover, the majority of the human population (undergraduates, graduates, and teachers) in campuses is well educated and generally more willing to protect wildlife [38]. Actually, biodiversity in campuses can also be used as an effective resource for education [39]. In this scenario, human population density may not be an appropriate surrogate for anthropogenic disturbance.

Moreover, campus management is also important for biodiversity conservation in university campuses. Although sometimes not directly targeted for wildlife conservation, biodiversity-friendly practices (e.g., ecological restoration and ecological planning projects) are common in university campuses, which also help to create more suitable habitats for ecological specialists and further alleviate the potentially negative effects of human population [40,41,42,43]. For example, Peking University has even set up a mini nature reserve within its main campus [37]. According to the relevant biodiversity management project, the whole campus was divided into four areas, namely biodiversity conservation areas, important species habitats, water bodies, and garden landscape areas [37]. Numerous volunteers (mostly students and teachers) have been involved with the project. These activities helped to provide ideal habitats for many organisms, including ecological specialists such as Common Kingfisher, Buff Browed Willow Warbler (*Phylloscopus armandii*), and Lesser Cuckoo (*Cuculus poliocephalus*).

Interestingly, the role of NDVI in shaping ecological specialization of bird communities was inconsistent. Generally, forests (or woodlands), grasslands, and lakes are the major habitats suitable for most birds in campuses. Compared to grasslands and lakes, forests and woodlands are usually associated with higher NDVI and more structurally complex habitats, providing more types of foraging stratums for birds. For example, forests are linked with at least four foraging stratums (foraging on ground, foraging in understory, foraging in mid to high levels in trees or high bushes, and foraging in or just above tree canopy), while grasslands only provide one foraging stratum (foraging on ground). However, forests or woodlands in university campuses do not necessarily provide more types of food resources than grasslands. Seeds, invertebrates, and vertebrates can be found in both habitats, and fruits generated by forests often drop to the ground and become available for birds foraging on grassland. For diet specialists foraging in the air (such as swallows and swifts), invertebrates (their major food) are also usually abundant above grasslands. Moreover, grasslands (or even bare ground) and lakes provide food for scavengers and species specialized to eating fish, respectively. Taken together, it is thus not surprising that NDVI was positively related to foraging stratum specialization but negatively related to diet specialization.

It should be noted that university campuses are somewhat different from other types of urban green spaces (such as parks), so that some conclusions drawn from our study may not directly apply to other types of urban green spaces. For example, the human populations in campuses are generally better educated and more unevenly distributed than those in parks. As a result, the relationship between human density and ecological specialization may also differ between university campuses and parks. Further investigations and comparative studies are required to understand whether and how ecological correlates of ecological specialization would differ among various types of urban green spaces.

Some limitations of the present study need to be mentioned. First, although we excluded some campuses that seemed to be inadequately sampled, information on actual sampling efforts of many campuses was not available. Therefore, our dataset may have involved some biases due to differences in sampling efforts, which may have caused biases in our results; second, although people in campuses are generally well educated and more likely to participate in protection or environment-friendly practices, human attitudes toward wildlife may also vary considerably among campuses. Such complexity might have also contributed to the spatial distribution of ecological specialization, but was not considered in the present study due to lack of data. Finally, long-term biodiversity monitoring is still scarce in Chinese university campuses, meaning we were unable to explore the temporal trends of specialization in avian communities within or across campuses. Actually, long-term ecological data (normally exceeding a decade) is necessary to understand the fundamental mechanisms of population dynamics, community succession, phenology, and evolution, which are crucial pieces of knowledge for both ecologists and conservation scientists. However, long-term field data collection is often difficult and many projects have been terminated early due to the lack of financial support [44]. In this scenario, we call for strengthening the role of citizen science in promoting long-term ecological research, which is not only useful in providing invaluable insights for scientific or conservation projects, but also helps to enhance residents’ incentives for environmental protection. Developing a systematic and consistent framework of campus biodiversity monitoring based on citizen science should be a good starting point.

## 5. Conclusions

In summary, our results suggest that university campuses with large areas or located at high altitudes tend to possess a higher level of avian specialization. Considering that ecological specialists are often more vulnerable to disturbance or climate change, university campuses may act as important refuges for these species, as well as a critical type of urban green space for biodiversity conservation. This is especially important when urbanization has been rapidly expanding worldwide, and more and more protection efforts need to be carried out in the context of human existence.

## Figures and Tables

**Table 1:** Results of generalized least square linear models on species richness, diet specialist richness, foraging stratum specialist richness, community-wide diet specialization index (diet CSI), and community-wide foraging stratum specialization index (foraging stratum CSI). For each explanatory variable, variance inflation factor (VIF) is indicated to reflect the level of multicollinearity.

Explanatory Variable	*Estimate*	*SE*	*Z* Value	*p* Value	*VIF*
Response variable: Species richness					
Intercept	10.907	3.719	2.933	0.004	N.A.
Campus area	7.071	1.047	6.752	<0.001	1.164
Elevation	0.005	0.002	2.969	0.003	1.021
NDVI	23.862	9.181	2.599	0.010	1.036
Human population density	−0.000	0.000	−0.620	0.536	1.179
Response variable: diet specialist richness					
Intercept	−2.246	0.516	−4.351	<0.001	N.A.
Species richness	0.232	0.010	23.151	<0.001	1.384
Campus area	−0.236	0.159	−1.489	0.138	1.454
Elevation	0.001	0.000	4.309	<0.001	1.070
NDVI	0.296	1.268	0.234	0.816	1.074
Human population density	0.000	0.000	2.485	0.014	1.181
Response variable: foraging stratum specialist richness					
Intercept	−0.482	0.459	−1.050	0.295	N.A.
Species richness	0.238	0.009	28.006	<0.001	1.370
Campus area	0.368	0.137	2.686	0.008	1.441
Elevation	0.000	0.000	0.084	0.933	1.068
NDVI	−1.688	1.127	−1.497	0.136	1.070
Human population density	0.000	0.000	0.062	0.951	1.178
Response variable: diet CSI					
Intercept	0.873	0.005	168.239	<0.001	N.A.
Species richness	0.001	0.000	6.139	<0.001	1.382
Campus area	−0.002	0.002	−1.313	0.191	1.452
Elevation	0.000	0.000	1.534	0.127	1.070
NDVI	−0.049	0.013	−3.860	<0.001	1.073
Human population density	0.000	0.000	0.240	0.810	1.181
Response variable: foraging stratum CSI					
Intercept	0.800	0.006	140.517	<0.001	N.A.
Species richness	0.000	0.000	1.045	0.298	1.379
Campus area	0.003	0.002	1.595	0.113	1.449
Elevation	0.000	0.000	0.513	0.609	1.070
NDVI	0.068	0.014	4.893	<0.001	1.072
Human population density	0.000	0.000	0.982	0.328	1.180

## Data Availability

Datasets used in this study are provided as Supplementary Materials.

## Supplementary Materials

The following supporting information can be downloaded at: https://www.mdpi.com/article/10.3390/biology14050570/s1, Table S1: Bird list; Table S2: Traits of birds; Table S3: Campus information.
